# Current perspectives on postoperative cognitive dysfunction in geriatric patients: insights from clinical practice

**DOI:** 10.3389/fmed.2024.1466681

**Published:** 2024-09-27

**Authors:** Liang Zhang, Yi Qiu, Zhi-feng Zhang, Yi-fan Zhao, Yu-mei Ding

**Affiliations:** ^1^Department of Anaesthesiology, The Second Affiliated Hospital of Inner Mongolia Medical University, Hohhot, China; ^2^Department of Joint Surgery, The Second Affiliated Hospital of Inner Mongolia Medical University, Hohhot, China; ^3^School of Public Health, Inner Mongolia Medical University, Hohhot, China

**Keywords:** postoperative cognitive dysfunction, geriatric, elderly, perspectives, insights

## Abstract

Postoperative cognitive dysfunction (POCD) is a common and serious postoperative complication in elderly patients, affecting cognitive function and quality of life. Its pathophysiology is complex, involving age-related cognitive decline, surgical and anesthetic factors, systemic and neuroinflammation, as well as genetic and environmental contributors. Comprehensive preoperative assessment and optimization, the selection of appropriate anesthetic agents, minimally invasive surgical techniques, and early postoperative rehabilitation and cognitive training are effective strategies to reduce the incidence of POCD. Recent research suggests that anti-inflammatory drugs and neuroprotective agents may be promising in preventing POCD. Additionally, non-pharmacological interventions, including cognitive and physical training, have shown positive effects. Future research directions should include large-scale clinical trials and mechanistic studies to further understand and manage POCD, along with integrating new findings into clinical practice. Continuous education and training for healthcare professionals are essential to ensure the effective application of the latest research findings in patient care. Through multidisciplinary collaboration and ongoing improvements, these efforts can significantly enhance the cognitive function and quality of life of elderly surgical patients.

## 1 Introduction

Postoperative cognitive dysfunction (POCD) refers to the decline in cognitive functions, including memory, attention, and executive function, observed in patients following anesthesia and surgery (1). This phenomenon is notably prevalent among elderly patients (2). The significance of POCD lies in its broad definition, encompassing various cognitive domains, and its substantial impact on patient recovery and long-term cognitive health (3). POCD is a significant clinical concern, especially among elderly surgical patients. Epidemiological data indicate that POCD affects about 30% of younger patients and 40% of older adults at the time of hospital discharge. This prevalence underscores a notable age-related susceptibility, with long-term cognitive deficits persisting in approximately 12.7% of elderly patients and 5% of younger patients 3 months post-surgery (4). POCD is common in high-risk surgical procedures, such as major cardiac, orthopedic, and vascular surgeries, where prolonged anesthesia and significant physiological stress are common contributing factors. These findings highlight the critical need for targeted preventive measures and management strategies to reduce the burden of POCD and improve patient outcomes across various surgical contexts.

The impact of POCD on patient outcomes and quality of life is multifaceted (1, 5). Cognitive decline can delay postoperative recovery, increase hospital stays and readmission rates, and impair daily living activities, thereby reducing patients’ ability to live independently after surgery (1). Additionally, POCD may elevate the risk of depression and anxiety, further deteriorating overall quality of life (6, 7). Therefore, preventing and managing POCD has become a critical focus in clinical medicine.

This study aims to provide an overview of current perspectives on POCD in elderly patients. We will review recent research findings, analyze the mechanisms and contributing factors of POCD in the elderly, explore its long-term impact on patient outcomes, and present current clinical insights and intervention strategies. By synthesizing existing literature, we seek to offer practical guidance for clinical practice and propose directions for future research. Through this study, we aim to enhance awareness of POCD among clinicians and researchers, promote the development of personalized intervention strategies for elderly patients, and ultimately improve postoperative cognitive function and quality of life for this population. This study also has significant implications for policymakers, providing a scientific basis for optimizing and implementing related health policies.

## 2 Pathophysiology of POCD in elderly patients

### 2.1 Age-related cognitive decline and vulnerability

As individuals age, they experience a natural decline in cognitive functions, such as memory, attention, and executive functioning (8). This decline results from neurobiological changes, including neuronal loss, synaptic degeneration, and reduced neuroplasticity (4, 8). Consequently, elderly patients have a diminished cognitive reserve, making them more susceptible to postoperative cognitive impairments (4). The aging brain’s increased vulnerability to external stressors, such as surgery and anesthesia, exacerbates pre-existing cognitive decline, thereby increasing the risk of POCD.

### 2.2 Surgery and anesthesia-related factors

#### 2.2.1 High-risk surgery types

Certain surgeries, notably major cardiac, orthopedic, and vascular procedures, are associated with a higher incidence of POCD (9–11). These surgeries typically involve prolonged durations, significant physiological stress, and extensive tissue trauma, all of which contributing to cognitive dysfunction (9–11). For example, cardiac surgeries can lead to cerebral microembolism and hypoperfusion, which directly impact cognitive function (10).

#### 2.2.2 Effects of different anesthetic agents

The type of anesthetic agent used significantly influences the development of POCD. General anesthesia, especially with volatile anesthetics, has been linked to higher POCD rates compared to regional anesthesia (12). Volatile anesthetics like isoflurane and sevoflurane have been shown to induce neuroinflammation and apoptosis in animal studies (13). In contrast, regional anesthesia, which limits systemic exposure to anesthetics and reduces overall physiological stress, may offer protective effects against POCD.

### 2.3 Inflammatory response and neuroinflammation

#### 2.3.1 Role of systemic inflammation

Surgery triggers a systemic inflammatory response characterized by the release of pro-inflammatory cytokines, such as IL-1β, IL-6, and TNF-*α* (14). These cytokines can cross the blood–brain barrier (BBB) and induce neuroinflammation, leading to neuronal damage and cognitive impairment (14). In elderly patients, dysregulated immune systems often result in an exaggerated and prolonged inflammatory response, which is particularly detrimental.

#### 2.3.2 BBB dysfunction

The integrity of the BBB is crucial for maintaining central nervous system homeostasis. Aging and surgical stress can compromise BBB function, increasing its permeability to inflammatory mediators and neurotoxic substances (15). This heightened permeability allows systemic inflammatory cytokines to infiltrate the brain, exacerbating neuroinflammation and contributing to POCD (16).

### 2.4 Structural and molecular basis of POCD and its clinical manifestations

The clinical manifestations of POCD, such as memory impairment, attention deficits, and executive dysfunction, are closely linked to structural and molecular changes in the brain. At the structural level, POCD is associated with neuronal loss, synaptic degeneration, and hippocampal atrophy, which directly impact cognitive functions like memory and learning. These structural changes are often exacerbated by disruptions in neuroplasticity and reduced synaptic density, both of which are critical for maintaining cognitive flexibility and function in the aging brain (15).

At the molecular level, the systemic inflammatory response triggered by surgery results in elevated levels of pro-inflammatory cytokines, such as IL-1β, IL-6, and TNF-*α*, which can penetrate the blood–brain barrier and promote neuroinflammation. This inflammatory cascade not only damages neurons but also alters synaptic function, contributing to the observed cognitive deficits in POCD (16). Furthermore, increased oxidative stress and the accumulation of neurotoxic proteins, such as amyloid-beta, have been implicated in synaptic dysfunction and neuronal apoptosis, further linking molecular disruptions to clinical symptoms.

These structural and molecular changes disrupt the integrity of neural networks involved in cognitive processes, thereby explaining the clinical manifestations observed in POCD. Understanding these connections is essential for developing targeted therapeutic strategies that address both the underlying pathophysiology and the clinical symptoms of POCD.

### 2.5 Genetic and environmental factors

#### 2.5.1 Genetic susceptibility

Genetic factors significantly influence an individual’s risk of developing POCD (17). Polymorphisms in genes related to inflammatory responses, such as the Apolipoprotein E (ApoE) ε4 allele, have been associated with a higher risk of POCD (17). Individuals carrying the ApoE ε4 allele are more prone to neuroinflammatory responses and subsequent cognitive decline post-surgery.

#### 2.5.2 Lifestyle and comorbidities

Environmental factors, including lifestyle and pre-existing comorbidities, further affect POCD risk. Lifestyle factors such as physical inactivity, poor diet, and smoking can impair cognitive reserve and exacerbate neuroinflammation (18). Common comorbidities in elderly patients, such as hypertension, diabetes, and cardiovascular diseases, also contribute to a higher risk of POCD (19, 20). These conditions lead to chronic low-grade inflammation, endothelial dysfunction, and cerebrovascular pathology, all of which are detrimental to cognitive health.

## 3 Clinical presentation and diagnosis

### 3.1 Clinical features

#### 3.1.1 Affected cognitive domains

The POCD primarily impacts cognitive domains such as memory, attention, and executive functions (3, 21). Memory impairment may present as difficulties in retaining and recalling new information, which can disrupt daily activities and social interactions. Attention deficits are characterized by reduced concentration, difficulty maintaining focus on tasks, and increased susceptibility to distractions. Executive function impairments involve challenges in planning, decision-making, and problem-solving. These cognitive deficits significantly impede a patient’s ability to return to normal life and work activities post-surgery (22).

#### 3.1.2 Timing and duration of symptoms

The onset and duration of POCD symptoms vary. Symptoms typically emerge within days to weeks post-surgery, with the acute phase usually occurring within the first week (23). While many patients experience transient cognitive decline that improves over weeks to months, some individuals may suffer from persistent deficits lasting for years (23). Chronic POCD is particularly concerning, as it can severely impact long-term quality of life and independence.

### 3.2 Diagnostic criteria and tools

#### 3.2.1 Neuropsychological testing and assessment

Diagnosing POCD requires comprehensive neuropsychological testing to evaluate various cognitive domains (10). Standardized tests, such as the Mini-Mental State Examination, Montreal Cognitive Assessment, and specific neuropsychological batteries (e.g., Wechsler Adult Intelligence Scale, Rey Auditory Verbal Learning Test) are employed to assess memory, attention, and executive functions (10, 24). Ideally, baseline cognitive assessments are conducted preoperatively to compare with postoperative scores, enabling the identification of significant cognitive declines.

#### 3.2.2 Biomarkers and imaging studies

Biomarkers and imaging studies increasingly support POCD diagnosis. Biomarkers such as elevated inflammatory cytokines (e.g., IL-6, TNF-*α*) and proteins related to neuronal injury (e.g., S100β, tau protein) in cerebrospinal fluid or blood may indicate neuroinflammation and neuronal damage (14, 25). Neuroimaging techniques, including magnetic resonance imaging (MRI) and positron emission tomography (PET), can reveal structural and functional brain changes associated with cognitive impairment (26). MRI can detect hippocampal atrophy and white matter lesions, while PET scans can show hypometabolism in brain regions involved in cognitive functions.

#### 3.2.3 Differential diagnosis

Differentiating POCD from other cognitive disorders, such as delirium and dementia is critical. Delirium is an acute, fluctuating disturbance of consciousness and cognition, typically occurring shortly after surgery and resolving within days, with prominent fluctuations in attention and awareness (27). Dementia is a progressive, persistent cognitive decline, usually unrelated to surgical events, involving significant impairments in daily functioning over months to years (27).

A thorough clinical evaluation, including detailed patient history, physical examination, and careful review of neuropsychological test results, helps distinguish POCD from these conditions (24). Monitoring cognitive changes over time and assessing potential contributing factors, such as medications, infections, and metabolic disturbances, are essential for accurate diagnosis (27).

In summary, the clinical presentation and diagnosis of POCD involve identifying specific cognitive deficits, assessing their onset and duration, and utilizing neuropsychological tests, biomarkers, and imaging studies. Accurate diagnosis is crucial for distinguishing POCD from other cognitive disorders and for developing targeted interventions to improve patient outcomes and quality of life post-surgery.

## 4 Prevention and management strategies

### 4.1 Preoperative assessment and optimization

#### 4.1.1 Comprehensive geriatric assessment

Conducting a comprehensive geriatric assessment (CGA) is essential for identifying elderly patients at risk for POCD. The CGA evaluates physical health, cognitive function, emotional status, social support, and functional abilities (28). This holistic assessment allows healthcare providers to pinpoint vulnerabilities and customize preoperative care to address specific needs (28). By establishing baseline cognitive function and overall health status, clinicians can develop personalized care plans to reduce the risk of POCD.

#### 4.1.2 Identification and modification of risk factors

Identifying and addressing modifiable risk factors before surgery can significantly reduce the incidence of POCD. Key risk factors include advanced age, pre-existing cognitive impairment, polypharmacy, and comorbidities such as cardiovascular disease, diabetes, and hypertension (18–20). Preoperative optimization may involve adjusting medications, managing chronic diseases, and correcting nutritional deficiencies. By minimizing these risk factors, healthcare providers can enhance patients’ resilience to surgical stress and improve postoperative outcomes.

#### 4.1.3 Prehabilitation and patient education

Prehabilitation, which encompasses physical exercise, nutritional optimization, and psychological support, can improve patients’ physical and mental resilience pre-surgery (29). Educating patients and their families about POCD, its potential impacts, and preventive measures can enhance engagement in preoperative and postoperative care (30). Empowering patients with knowledge and strategies to manage stress and maintain cognitive health can help reduce anxiety and promote a smoother recovery.

#### 4.1.4 Challenges and deficiencies in perioperative management of POCD

Although there have been advancements in managing POCD, significant challenges and deficiencies remain in clinical practice. A primary challenge is the absence of standardized, evidence-based guidelines for the perioperative management of POCD, resulting in considerable variability in care practices across healthcare settings. This inconsistency can lead to disparate patient outcomes and a lack of consensus on best practices for prevention and treatment (31). Additionally, there is a widespread deficiency in the routine use of CGA and cognitive screening tools during the preoperative phase (32). These tools are crucial for identifying patients at heightened risk of POCD, yet they are often underutilized due to time constraints, resource limitations, or a lack of awareness among healthcare providers. This gap in early assessment can prevent the implementation of tailored interventions that might mitigate the risk of cognitive decline.

The integration of multidisciplinary care remains another significant challenge. Optimal management of POCD requires coordination among anesthesiologists, surgeons, geriatricians, neurologists, and rehabilitation specialists (33). However, the lack of structured collaboration between these disciplines often results in fragmented care and missed opportunities for comprehensive perioperative management. Furthermore, the current reliance on pharmacological interventions without sufficient emphasis on non-pharmacological strategies, such as cognitive training, physical rehabilitation, and patient education, reflects a gap in holistic patient care. Non-pharmacological interventions have demonstrated potential in reducing the incidence and severity of POCD, but their incorporation into standard perioperative protocols is inconsistent.

Addressing these challenges necessitates the development of robust, multidisciplinary guidelines, enhanced training programs for healthcare providers, and the adoption of a more integrative approach that combines both pharmacological and non-pharmacological strategies.

### 4.2 Intraoperative techniques

#### 4.2.1 Anesthetic considerations

Selecting appropriate anesthetic agents and monitoring anesthesia depth are critical to reducing the risk of POCD. Regional anesthesia is often preferred for elderly patients due to its lower impact on cognitive function (34). When general anesthesia is necessary, agents with shorter half-lives and minimal neurotoxic effects, such as propofol, should be used (34). Continuous monitoring of anesthesia depth using technologies like the bispectral index helps avoid over-sedation and minimizes cognitive side effects (35).

#### 4.2.2 Minimally invasive surgical techniques

Minimally invasive surgical techniques, such as laparoscopic or robotic-assisted surgery, reduce physiological stress, tissue trauma, and inflammatory response compared to traditional open surgeries (36). These approaches are associated with shorter operative times, reduced postoperative pain, and faster recovery, all of which contribute to a lower risk of POCD (37). By minimizing surgical invasiveness, healthcare providers can improve postoperative cognitive outcomes in elderly patients.

#### 4.2.3 Perioperative hemodynamic and glycemic management

Maintaining stable hemodynamic parameters and optimal blood glucose levels during surgery is crucial for preventing POCD (38, 39). Hypotension, hypoxia, and hyperglycemia can exacerbate neuroinflammation and cognitive decline (38, 39). Close monitoring and timely intervention to maintain adequate cerebral perfusion and oxygenation, along with strict glycemic control, are vital strategies (39). Implementing standardized protocols for perioperative hemodynamic and glycemic management can improve overall surgical outcomes and cognitive function.

### 4.3 Postoperative care

#### 4.3.1 Early mobilization and rehabilitation

Early mobilization and rehabilitation are vital for mitigating POCD (40). Encouraging patients to engage in light physical activities soon after surgery enhances blood circulation, reduces inflammation, and promotes cognitive recovery (40). Structured rehabilitation programs tailored to patients’ capabilities accelerate functional recovery and improve cognitive outcomes (21).

#### 4.3.2 Pain management and delirium prevention

Effective pain management is crucial in preventing delirium and subsequent cognitive decline (41). Multimodal analgesia, combining different pain relief strategies, can minimize opioid use and its cognitive side effects. Additionally, implementing protocols to prevent delirium—such as maintaining a calm and oriented environment, optimizing sleep, and managing sensory deficits—is essential to reduce the risk of POCD (41).

#### 4.3.3 Cognitive training and support programs

Postoperative cognitive training and support programs can aid patients in recovering cognitive functions and maintaining mental agility. Cognitive exercises, such as memory games, problem-solving tasks, and attention training, stimulate neuroplasticity and enhance cognitive recovery (42). Support programs, including counseling and social engagement activities, provide emotional and social support, contributing to overall well-being and cognitive health (43).

In summary, a multifaceted approach involving preoperative assessment and optimization, intraoperative techniques, and comprehensive postoperative care is essential for preventing and managing POCD in elderly patients. By implementing these strategies, healthcare providers can improve cognitive outcomes and enhance the quality of life for elderly patients undergoing surgery.

## 5 Recent advances and research findings

### 5.1 Pharmacological approaches

Recent research has focused on developing pharmacological interventions to mitigate POCD. One promising avenue involves anti-inflammatory drugs, as systemic and neuroinflammation play critical roles in POCD pathogenesis (44). Non-steroidal anti-inflammatory drugs (NSAIDs) and corticosteroids are under investigation for their potential to reduce inflammation and improve cognitive outcomes post-surgery (44, 45). For example, perioperative administration of dexamethasone has shown promise in reducing inflammatory markers and preserving cognitive function in elderly surgical patients.

Neuroprotective agents are another area of interest (46). Drugs such as acetylcholinesterase inhibitors, traditionally used in Alzheimer’s disease treatment, are being evaluated for their efficacy in preventing POCD (46). These agents may enhance cholinergic transmission, thereby supporting cognitive function during the postoperative period. Additionally, antioxidants like melatonin and vitamins E and C are being studied for their potential to protect neural tissue from oxidative stress associated with surgery and anesthesia.

### 5.2 Non-pharmacological strategies

Non-pharmacological interventions also hold significant promise in managing POCD (47). Cognitive therapy, including cognitive-behavioral interventions and cognitive training programs, aims to enhance cognitive resilience and function (47). These therapies can be tailored to individual patient needs, focusing on improving specific cognitive domains affected by POCD (47). For instance, cognitive training exercises targeting memory, attention, and executive functions have been shown to improve cognitive outcomes in elderly patients post-surgery.

Physical exercise is another effective non-pharmacological strategy (47). Regular physical activity has been demonstrated to improve overall brain health, enhance neuroplasticity, and reduce inflammation (47). Structured exercise programs initiated preoperatively and continued postoperatively can help maintain cognitive function and accelerate recovery. Combining physical exercise with cognitive therapy may offer synergistic benefits, further enhancing cognitive recovery in elderly surgical patients.

### 5.3 Emerging trends in POCD research

Recent trends in POCD research emphasize a multidisciplinary approach to prevention and management (48). This approach involves combining pharmacological treatments with lifestyle modifications, such as diet and exercise, to create a holistic strategy for cognitive health (48, 49). Researchers are also focusing on identifying biomarkers that can predict POCD risk, allowing for early intervention and personalized treatment plans.

Advances in neuroimaging and genetic profiling are providing deeper insights into the mechanisms underlying POCD. For instance, advanced MRI techniques are being used to study structural brain changes associated with POCD (50), while genetic studies explore the role of specific genes in susceptibility to cognitive decline post-surgery (17). These innovative research approaches are paving the way for more targeted and effective interventions.

In summary, significant progress has been made in understanding and managing POCD. Both pharmacological and non-pharmacological interventions show promise in mitigating cognitive decline in elderly surgical patients. Emerging research trends highlight the importance of a comprehensive, multidisciplinary approach to POCD prevention and management, offering hope for improved cognitive outcomes and quality of life for elderly patients.

## 6 Clinical studies and insights

### 6.1 Clinical study overviews

#### 6.1.1 Clinical studies of POCD in elderly patients

The POCD in elderly patients remains a critical concern in surgical outcomes, with numerous clinical studies exploring various interventions to mitigate its occurrence (13, 51–73). These studies range from observational to randomized controlled trials, providing comprehensive insights into the efficacy of different treatments (13, 51–73) (Table 1).

**Table 1:** Clinical studies of postoperative cognitive dysfunction in geriatric patients.

Study ref	Publication type	Disease	Sample size	Treatment	Main findings
Wang et al. (51)	Observational study	Elderly patients with TKA	40	TEAS	Combining conventional intraoperative nursing with TEAS can reduce POCD by reducing inflammation and neuronal injury
Mi et al. (52)	Randomized controlled trial	Elderly patients with metabolic syndrome	116	Intranasal insulin	Intranasal insulin reduces POCD and alleviates peripheral inflammation in elderly patients with metabolic syndrome
Takazawa et al. (53)	Randomized controlled trial	Elderly patients with TKA	202	Minocycline	Minocycline is unlikely to prevent POCD
Wang et al. (54)	Randomized controlled trial	Elderly patients	100	DEX	DEX reduced POCD and inflammation in elderly patients undergoing intubation
Olotu et al. (55)	Randomized controlled trial	Elderly patients with cardiovascular surgery	609	Delirium preventive measures	An intervention combining measures from established postoperative delirium prevention programs did not reduce POCD rates in older adults
Yang et al. (56)	Retrospective study	Elderly patients with rheumatic heart valve disease	100	Combined Etomidate-Ketamine anesthesia	Etomidate-ketamine anesthesia improves ECG stability, cognitive function, and pain in elderly heart valve patients
Xi et al. (57)	Randomized controlled trial	Elderly patients with gastrointestinal tumor	68	TEAS	Perioperative TEAS reduces inflammation and POCD while improving cognitive function in elderly gastrointestinal tumor patients
Orhun et al. (58)	Randomized controlled trial	Elderly patients	116	Epidural analgesia combined with GA	Combined GA and epidural analgesia result in similar POCD rates as GA alone, but better preserve memory, language, and visuospatial functions, likely due to effective pain control
Quan et al. (59)	Randomized controlled trial	Elderly patients undergoing abdominal surgery	120	BIS-guided deep anesthesia	Deep total intravenous anesthesia during abdominal surgery in elderly patients may reduce short-term POCD and peripheral inflammation postoperatively compared to light anesthesia
Li et al. (60)	Randomized controlled trial	Elderly patients	164	Propofol, DEX, and midazolam	Propofol sedation shows significant short-term advantages over DEX and midazolam sedation in elderly patients regarding POCD incidence
Langer et al. (61)	Randomized controlled trial	Elderly patients undergoing GA for surgery	101	Monitoring intraoperative hypotension	Intraoperative hypotension did not correlate with POCD or delirium, necessitating a multicenter trial to confirm its impact on POCD
Lu et al. (62)	Randomized controlled trial	Elderly patients after shoulder arthroscopy	152	Parecoxib sodium and DEX	Pretreatment with parecoxib sodium combined with DEX may reduce early POCD in elderly patients, potentially by improving postoperative analgesia and cerebral oxygen metabolism
Zhang et al. (63)	Randomized controlled trial	Elderly patients	90	EA	Preconditioning with EA could enhance postoperative cognitive function, possibly through mitigating inflammatory reactions and brain injury mechanisms
Sun et al. (64)	Randomized controlled trial	Elderly patients during hip-joint replacement surgery	300	Intravenous methoxamine infusion	Methoxamine infusion at 2–3 μg·kg^−1^·min^−1^ stabilizes hemodynamics without increasing POCD; 4 μg·kg^−1^·min^−1^ offers no benefits and may adversely affect intraoperative hemodynamics
Chi et al. (65)	Randomized controlled trial	Elderly patients	142	GA	Preoperative scopolamine butylbromide injection is clinically significant for POCD prevention, correlating with elevated S100b protein and plasma neuron-specific enolase levels in patient serum, indicating disease severity
Geng et al. (13)	Randomized controlled trial	Elderly patients undergoing laparoscopic cholecystectomy	150	Propofol, sevoflurane, and isoflurane	Propofol anesthesia may be an option for elderly surgical patients
Zhu et al. (66)	Randomized controlled trial	Elderly patients with TKA	134	Parecoxib	Parecoxib sodium reduces POCD occurrence post TKA in elderly patients by attenuating inflammation and acute postoperative pain associated with surgical trauma
Qiao et al. (67)	Randomized controlled trial	Elderly patients undergoing major surgery	90	IHA	Elderly surgery patients had higher POCD rates with sevoflurane IHA versus propofol intravenous maintenance, but lower with methylprednisolone pre-treatment, with elevated plasma S-100β, TNF-α, and IL-6 levels in sevoflurane recipients
Chen et al. (68)	Randomized controlled trial	Elderly patients following spine surgery	80	Intravenous lidocaine	Lidocaine may be effective in treating early POCD in elderly patients undergoing spine surgery
Tang et al. (69)	Randomized controlled trial	Elderly patients with MCI undergoing radical rectal resection	200	IHA	POCD incidence 7 days post radical rectal resection was similar with sevoflurane or propofol anesthesia, but sevoflurane had a greater cognitive impact than propofol in elderly MCI patients
Sun et al. (70)	Randomized controlled trial	Elderly patients	124	Dobutamine hydrochloride	The plasma concentration of TNFα was implicated in the effect of dobutamine hydrochloride on POCD
Rossi et al. (71)	Randomized controlled trial	Elderly patients	79	Serum anticholinergic activity	POCD is likely not primarily caused by perioperative anticholinergic medications but rather by other mechanisms
Fujita et al. (72)	Randomized controlled trial	Elderly patients undergoing upper abdominal surgery	20	Different perioperative analgesic methods	Perioperative analgesia with intravenous remifentanil and epidural ropivacaine demonstrated comparable incidence rates of early POCD following upper abdominal surgery in elderly patients
Cai et al. (73)	Randomized controlled trial	Elderly patients undergoing intravenous anesthesia and IHA	2000	Apolipoprotein E4 association	There was a strong association between apolipoprotein E ε4 and postoperative cognitive dysfunction in elderly patients undergoing IHA

TKA, total knee arthroplasty; TEAS, transcutaneous electrical acupoint stimulation; DEX, dexmedetomidine; ECG, electrocardiogram; TEAS, transcutaneous electrical acupoint stimulation; BIS, bispectral index; EA, electro-acupuncture; MCI, mild cognitive impairment; GA, general anesthesia; IHA, inhalation anesthesia.

#### 6.1.2 Clinical management and outcomes

Non-pharmacological interventions such as transcutaneous electrical acupoint stimulation (TEAS) have shown promise in reducing POCD. Wang et al. (51) demonstrated that combining conventional nursing with TEAS significantly reduced POCD by decreasing inflammation and neuronal injury in elderly patients undergoing total knee arthroplasty (TKA). Similarly, Xi et al. (57) found that perioperative TEAS reduced inflammation and POCD while improving cognitive function in elderly gastrointestinal tumor patients. Zhang et al. (63) further supported this by showing that preconditioning with electro-acupuncture (EA) enhanced postoperative cognitive function by mitigating inflammatory reactions and brain injury. However, Olotu et al. (55) noted that delirium preventive measures did not significantly lower POCD rates in older adults undergoing cardiovascular surgery, suggesting that tailored interventions are necessary.

Pharmacological interventions have also been extensively studied. Mi et al. (52) reported that intranasal insulin reduced POCD and alleviated peripheral inflammation in elderly patients with metabolic syndrome undergoing non-cardiac surgery. Takazawa et al. (53) evaluated the neuroprotective potential of minocycline in elderly patients after TKA but found no significant efficacy in preventing POCD. Wang et al. demonstrated that dexmedetomidine (DEX) anesthesia reduced both POCD and inflammation in elderly patients (55) (Table 1). Other studies explored the effects of different pharmacological agents: Lu et al. (62) found that parecoxib sodium pretreatment combined with dexmedetomidine reduced early POCD by improving postoperative analgesia and cerebral oxygen metabolism in elderly patients after shoulder arthroscopy, while Zhu et al. (66) showed that parecoxib sodium reduced POCD occurrence post-TKA by attenuating inflammation and acute postoperative pain. Sun et al. (64) reported that intravenous methoxamine infusion stabilized hemodynamics without increasing POCD during hip-joint replacement surgery. Chen et al. (68) found that intravenous lidocaine was effective in treating early POCD following spine surgery. Sun et al. (70) highlighted the role of dobutamine hydrochloride, which influenced POCD development through the plasma concentration of TNF-*α*. Chi et al. (65) noted the clinical significance of preoperative scopolamine butylbromide injection in POCD prevention in elderly patients undergoing general anesthesia (GA), and Geng et al. (13) suggested that propofol anesthesia might be preferable for elderly patients undergoing laparoscopic cholecystectomy compared to sevoflurane or isoflurane. Qiao et al. (67) observed higher POCD rates with sevoflurane inhalational anesthesia compared to propofol intravenous maintenance in elderly patients undergoing major surgery. Tang et al. (69) evaluated the impact of inhalational anesthetic on POCD in elderly patients with mild cognitive impairment undergoing radical rectal resection. Li et al. (60) found that propofol showed significant short-term advantages over DEX and midazolam in reducing POCD incidence. Langer et al. (61) concluded that intraoperative hypotension did not correlate with POCD and suggested the need for larger multicenter trials. Rossi et al. (71) suggested that POCD is likely not primarily caused by perioperative anticholinergic medications but rather by other mechanisms. Fujita et al. (72) found comparable incidence rates of early POCD with intravenous remifentanil and epidural ropivacaine in elderly patients undergoing upper abdominal surgery. Cai et al. (73) emphasized the strong association between apolipoprotein E ε4 and POCD, indicating the need for genetic considerations in managing cognitive outcomes post-surgery.

Combined interventions have also been investigated. Yang et al. (56) demonstrated that combined etomidate-ketamine anesthesia improved cognitive function and perioperative ECG stability in elderly patients with rheumatic heart valve disease. Quan et al. (59) showed that BIS-guided deep anesthesia during abdominal surgery reduced short-term POCD and peripheral inflammation. Orhun et al. (58) found that combined general anesthesia and epidural analgesia resulted in similar POCD rates compared to general anesthesia alone but better preserved memory, language, and visuospatial functions due to effective pain control.

These studies collectively underscore the multifaceted approaches to managing POCD in elderly patients. Effective clinical management requires a nuanced understanding of individual patient needs, surgical contexts, and the potential benefits of various interventions, from pharmacological treatments to non-pharmacological techniques like TEAS.

### 6.2 Lessons learned

#### 6.2.1 Best practices and common pitfalls

Several best practices have emerged from clinical studies on POCD. First, thorough preoperative cognitive assessments enable early identification of at-risk patients and the development of individualized care plans (74). Second, selecting appropriate anesthetic agents and ensuring adequate intraoperative monitoring can reduce neuroinflammation and cognitive decline (75). Third, early postoperative rehabilitation, including physical and cognitive exercises, is crucial for promoting cognitive recovery (21).

However, there are common pitfalls that must be addressed to improve outcomes. Inadequate preoperative assessments can lead to underestimation of POCD risk, while insufficient intraoperative monitoring may result in preventable cognitive declines. Additionally, delaying postoperative rehabilitation can hinder recovery, emphasizing the need for timely interventions. Avoiding these pitfalls requires adherence to established protocols and continuous evaluation of patient care practices.

#### 6.2.2 Multidisciplinary team insights

A multidisciplinary approach is essential for effectively managing POCD. Insights from various specialties, including anesthesiology, geriatrics, neurology, and physical therapy, contribute to comprehensive care strategies (76). Anesthesiologists can optimize anesthesia plans and monitor intraoperative parameters, while geriatricians provide expertise in managing age-related risk factors (77).

Neurologists assist in diagnosing and differentiating POCD from other cognitive disorders, ensuring accurate treatment (78). Physical therapists design and implement rehabilitation programs tailored to individuals’ needs (79). Collaboration among these specialists fosters a holistic approach, addressing the multifaceted nature of POCD and improving patient outcomes.

#### 6.2.3 Impact on clinical practice and integration of clinical experiences

This review highlights the significance of POCD management in clinical practice, emphasizing the need for the systematic integration of current evidence into everyday perioperative care (80). One of the main recommendations is the adoption of standardized cognitive screening and comprehensive risk assessment protocols, which are essential for identifying high-risk patients early (80). Implementing these protocols can enable healthcare providers to initiate timely interventions, ultimately reducing the prevalence and severity of POCD and enhancing patient recovery and quality of life. Furthermore, the review underscores the value of leveraging insights from previous clinical experiences to refine management strategies for POCD (81). Evidence suggests that multidisciplinary approaches, involving collaboration among anesthesiologists, geriatricians, surgeons, and neurologists, offer a more robust framework for managing the diverse aspects of POCD. Drawing on past clinical cases and outcomes can help in developing more cohesive and effective care pathways, ensuring that interventions are comprehensive and well-coordinated.

To effectively translate research into clinical practice, it is crucial to develop clear, evidence-based guidelines that detail best practices for POCD prevention and management (82). These guidelines should be complemented by ongoing professional development programs, including training and education for healthcare providers on the latest advancements and practical approaches to POCD care. Disseminating successful case studies and intervention models can also provide practical examples, aiding clinicians in implementing research findings within their own practice settings.

Additionally, the integration of feedback from clinical practice is vital for the continuous improvement of POCD management protocols (83). An iterative approach, in which protocols are regularly reviewed and adjusted based on clinical outcomes and new research, ensures that care strategies remain current and effective. This dynamic process not only helps in addressing any challenges encountered in practice but also supports the evolution of POCD management to better meet the needs of patients. By fostering a culture of evidence-based practice and continuous quality improvement, healthcare providers can enhance the overall standard of care for patients at risk of POCD.

## 7 Future directions and research

### 7.1 Emerging therapies and interventions

#### 7.1.1 Novel pharmacological treatments

The development of new pharmacological treatments for POCD is a promising area of research (84). Future studies are exploring agents aimed at reducing neuroinflammation, oxidative stress, and neuronal damage associated with surgery. Anti-inflammatory drugs targeting specific cytokines involved in neuroinflammation are a key focus (85). Additionally, neuroprotective agents that support synaptic function, and neuronal survival are being investigated. These include drugs that modulate neurotransmitter systems, antioxidants that counteract oxidative damage, and compounds that enhance neuroplasticity. As our understanding of the molecular pathways involved in POCD deepens, these novel pharmacological interventions could provide targeted and effective treatments.

#### 7.1.2 Non-pharmacological approaches

Non-pharmacological interventions, such as cognitive training and physical exercise, remain vital areas of research (47). Cognitive training programs designed to enhance memory, attention, and executive functions can help improve cognitive resilience and recovery in elderly surgical patients (47). These programs are often personalized to address specific deficits identified in preoperative assessments. Physical exercise, with its benefits on overall brain health and neuroplasticity, is also a key focus (47). Research is ongoing to determine the most effective types and intensities of exercise for supporting cognitive function postoperatively. Combining these non-pharmacological strategies with pharmacological treatments may offer a comprehensive approach to managing POCD.

#### 7.1.3 Optimizing existing protocols and exploring new research directions

Enhancing the management of postoperative cognitive dysfunction (POCD) requires a strategic approach to optimizing existing medical protocols and exploring novel research avenues. A primary step in optimizing current protocols involves the standardization of perioperative cognitive assessments, such as CGA and routine cognitive screening tools (86). The standardized use of these assessments can enable early identification of patients at elevated risk for POCD, facilitating the implementation of personalized and targeted preventive strategies.

Improvements in anesthetic management, including the careful selection of anesthetic agents with lower neurotoxic potential and the use of advanced monitoring technologies, can also reduce the risk of POCD (86). Incorporating depth of anesthesia monitoring and cerebral oximetry into standard practice allows for real-time adjustments, thereby minimizing perioperative factors that contribute to cognitive decline (86). Research into refining anesthetic protocols should prioritize agents and techniques that demonstrate a protective effect on cognitive function, especially in vulnerable populations.

Expanding the integration of non-pharmacological interventions, such as cognitive prehabilitation, physical exercise, and early mobilization, into perioperative care protocols is another critical area for optimization (29). These interventions have shown promise in reducing the incidence and severity of POCD but are not yet widely adopted. Future research should aim to establish robust evidence through large-scale clinical trials, which can validate the effectiveness of these strategies and support their incorporation into routine clinical guidelines.

New research directions should focus on the discovery of biomarkers for early detection and risk stratification of POCD, which would facilitate the development of personalized treatment plans. Additionally, exploring the roles of the gut-brain axis, neuroinflammation, and genetic predispositions in POCD could reveal novel therapeutic targets. Understanding these complex interactions may lead to breakthroughs in the prevention and management of POCD, moving beyond conventional approaches to more precise and individualized care.

To advance these efforts, a coordinated, multidisciplinary approach is essential. This includes fostering collaboration among anesthesiologists, surgeons, geriatricians, neurologists, and other specialists involved in perioperative care. Establishing comprehensive, evidence-based guidelines and enhancing the education and training of healthcare providers will be pivotal in translating research findings into clinical practice, ultimately improving patient outcomes in the management of POCD.

### 7.2 Further research areas

#### 7.2.1 Mechanistic studies of POCD

Understanding the precise mechanisms underlying POCD is crucial for developing effective interventions (87). Future research should focus on elucidating the molecular and cellular pathways contributing to cognitive decline following surgery. This includes studying the roles of neuroinflammation, blood–brain barrier dysfunction, and genetic susceptibility (87). Advanced neuroimaging techniques and biomarker analyses will be instrumental in these investigations, providing insights into the brain changes associated with POCD. Such mechanistic studies are essential for identifying new therapeutic targets and improving our overall understanding of POCD pathophysiology.

#### 7.2.2 Large-scale clinical trials

Conducting large-scale clinical trials is essential to validate the efficacy of emerging therapies and interventions for POCD (32). These trials should aim to enroll diverse populations to ensure the generalizability of findings. Key areas of focus include the timing, dosage, and combination of pharmacological and non-pharmacological treatments. Additionally, trials should also investigate the long-term effects of these interventions on cognitive function and quality of life (32). Large-scale studies will provide the robust evidence needed to translate research findings into clinical practice and inform guidelines for POCD management.

### 7.3 Impact on clinical practice

#### 7.3.1 Integrating new findings into practice

Incorporating the latest research findings into clinical practice is crucial for improving the management of POCD (88). This involves updating clinical guidelines and protocols to reflect evidence-based interventions. For instance, standardized preoperative cognitive assessments and tailored perioperative care plans should become routine practice. Clinicians need to stay informed about the latest pharmacological and non-pharmacological treatments and consider these options when planning patient care. Integrating new research findings into clinical practice will require collaboration between researchers and healthcare providers to ensure that advancements are effectively translated into patient care.

#### 7.3.2 Continuous education and training of healthcare professionals

Ongoing education and training for healthcare professionals are vital for the successful implementation of new POCD management strategies (89). This includes providing training on using cognitive assessment tools, understanding the mechanisms of POCD, and applying evidence-based interventions. Continuing medical education programs, workshops, and interdisciplinary team meetings can facilitate knowledge transfer and skill development (90). Ensuring that all members of the healthcare team are well-informed about the latest research and best practices will enhance the quality of care for elderly surgical patients and reduce the incidence of POCD.

In summary, the future directions and research in POCD encompass emerging therapies, further mechanistic studies, and the integration of new findings into clinical practice. By focusing on these areas, the medical community can develop more effective strategies to prevent and manage POCD, ultimately improving outcomes and quality of life for elderly patients undergoing surgery.

### 7.4 Implications and future directions

The findings of this review highlight several implications for clinical practice and future research. First, the standardization of preoperative cognitive screening, such as the use of CGA, is critical for identifying patients at risk for POCD and enabling early, targeted interventions (86). This approach can help tailor perioperative care to individual patient needs, potentially reducing the incidence and severity of cognitive dysfunction.

Second, optimizing anesthetic protocols to include agents with lower neurotoxicity and utilizing advanced monitoring tools, such as cerebral oximetry and depth of anesthesia monitoring, can play a pivotal role in minimizing the cognitive impact of surgery (80). Future research should explore the comparative effectiveness of various anesthetic techniques and agents, particularly in elderly populations.

Additionally, the integration of non-pharmacological interventions—such as cognitive training, early mobilization, and structured physical rehabilitation—into perioperative care should be prioritized (91). These strategies have demonstrated efficacy in enhancing cognitive resilience, yet their implementation remains inconsistent in clinical practice. Large-scale clinical trials are needed to validate these interventions and inform comprehensive guidelines for their use.

Finally, advancing research into novel biomarkers, neuroinflammatory pathways, and genetic predispositions associated with POCD will support the development of personalized medicine approaches. Understanding the underlying mechanisms of POCD can lead to the identification of new therapeutic targets, fostering more effective and individualized treatments. A collaborative, multidisciplinary approach involving anesthesiologists, geriatricians, neurologists, and rehabilitation specialists is essential to translating these research findings into practice, ultimately improving perioperative care and patient outcomes.

## 8 Summary

This study underscores the substantial impact of POCD on the cognitive health and overall recovery of elderly surgical patients. It highlights key strategies, including the routine implementation of standardized cognitive assessments, optimization of anesthetic protocols, and the integration of both pharmacological and non-pharmacological interventions to prevent and manage POCD. By prioritizing these evidence-based approaches, clinical practices can evolve to provide more personalized and effective care, thereby enhancing patient outcomes and quality of life post-surgery. Future research should continue to refine these strategies and explore novel therapeutic targets to further reduce the burden of POCD.

## Data Availability

The original contributions presented in the study are included in the article/supplementary material, further inquiries can be directed to the corresponding authors.
